# Arginine as an environmental and metabolic cue for cyclic diguanylate signalling and biofilm formation in *Pseudomonas putida*

**DOI:** 10.1038/s41598-020-70675-x

**Published:** 2020-08-12

**Authors:** Laura Barrientos-Moreno, María Antonia Molina-Henares, María Isabel Ramos-González, Manuel Espinosa-Urgel

**Affiliations:** 1 Department of Environmental Protection, Estación Experimental del Zaidín, CSIC, Granada, Spain; 2 Present Address: National Biofilms Innovation Centre, Biodiscovery Institute, School of Life Sciences, University of Nottingham, Nottingham, NG7 2RD UK

**Keywords:** Biofilms, Microbial genetics

## Abstract

Cyclic diguanylate (c-di-GMP) is a broadly conserved intracellular second messenger that influences different bacterial processes, including virulence, stress tolerance or social behaviours and biofilm development. Although in most cases the environmental cue that initiates the signal transduction cascade leading to changes in cellular c-di-GMP levels remains unknown, certain l- and d-amino acids have been described to modulate c-di-GMP turnover in some bacteria. In this work, we have analysed the influence of l-amino acids on c-di-GMP levels in the plant-beneficial bacterium *Pseudomonas putida* KT2440, identifying l-arginine as the main one causing a significant increase in c-di-GMP. Both exogenous (environmental) and endogenous (biosynthetic) l-arginine influence biofilm formation by *P. putida* through changes in c-di-GMP content and altered expression of structural elements of the biofilm extracellular matrix. The contribution of periplasmic binding proteins forming part of amino acid transport systems to the response to environmental l-arginine was also studied. Contrary to what has been described in other bacteria, in *P. putida* these proteins seem not to be directly responsible for signal transduction. Rather, their contribution to global l-arginine pools appears to determine changes in c-di-GMP turnover. We propose that arginine plays a connecting role between cellular metabolism and c-di-GMP signalling in *P. putida*.

## Introduction

Cyclic nucleotides are important signalling molecules in both prokaryotes and eukaryotes, with diverse functions as second messengers. In many bacterial species, the intracellular second messenger cyclic diguanylate (c-di-GMP) plays a key role in the transition between planktonic and sessile lifestyles: high levels of this molecule generally favour bacterial adhesion to surfaces and the establishment of biofilms, whereas low levels promote biofilm dispersal^1,2^ The enzymatic activities diguanylate cyclase (DGC) and phosphodiesterase (PDE) are responsible for the synthesis and degradation of this signal molecule, respectively^1,3^.

In the plant-beneficial bacterium *Pseudomonas putida* KT2440, the gene *cfcR* encodes a response regulator with DGC activity that has been characterized in detail. It was first identified as preferentially expressed in bacterial populations associated to plant roots^4^, and later shown to increase c-di-GMP levels and give rise to a pleiotropic phenotype when cloned in *P. putida* in a multicopy plasmid under the control of its own promoter^5^. This phenotype includes increased biofilm formation, cell flocculation in liquid cultures, pellicle formation in the air–liquid interface, and altered (crinkly) colony morphology in solid medium. Expression of *cfcR* is subject to a complex, multi-level control that involves global regulators, including the stationary phase sigma factor RpoS^5^ and the three post-transcriptional regulators of the CsrA/RsmA family identified in KT2440, RsmA, RsmE and RsmI^6^.

In a previous high throughput analysis, it was shown that transposon mutations in *argG* and *argH*, the genes encoding the last two enzymes in the arginine biosynthesis pathway, abolish the crinkly colony morphology phenotype associated to the presence of the plasmid harbouring *cfcR*^7^. This phenotype loss is associated to reduced c-di-GMP levels in those mutants despite the presence of *cfcR* in multicopy, and addition of l-arginine to the growth medium restored the crinkly colony morphology in the mutants and increased c-di-GMP levels in the wild type^7^. However, the mechanism underlying this connection between arginine metabolism and c-di-GMP signalling in *P. putida* remained to be established. Previous reports have shown that l-arginine increases biofilm formation and represses swarming motility in *Pseudomonas aeruginosa* PA14, an effect that requires the presence of functional DGCs SadC and/or RoeA^8^. Similarly, l-arginine has been found to induce the synthesis of c-di-GMP in *Salmonella enterica* serovar Typhimurium, through the periplasmic arginine-binding protein ArtI and the DGC STM1987, which also contains a periplasmic domain^9^. However, a survey of its genome indicates that no homolog of this DGC can be found in *P. putida*. This, and the fact that c-di-GMP levels in this bacterium appear to respond to intracellular pools of arginine resulting from the biosynthetic pathway, as well as to exogenously added amino acid^7^, suggested the existence of a different signalling circuit from that proposed in *Salmonella*. This apparent evolutionary convergence in a signal response makes its study of particular interest.

In this work we present further evidence on the role of l-arginine as both an environmental and a metabolic signal that modulates the lifestyles of *P. putida* through c-di-GMP signalling and changes in the expression of biofilm matrix components. Our data indicate that different periplasmic amino acid-binding proteins, each associated to a transport system, participate in different ways in the response to external l-arginine, and suggest that the synthesis of the second messenger c-di-GMP is modulated by the state of global arginine pools resulting from anabolism as well as uptake.

## Results

### l-Arginine increases c-di-GMP levels and promotes biofilm formation in *P. Putida*.

**Figure 1:**
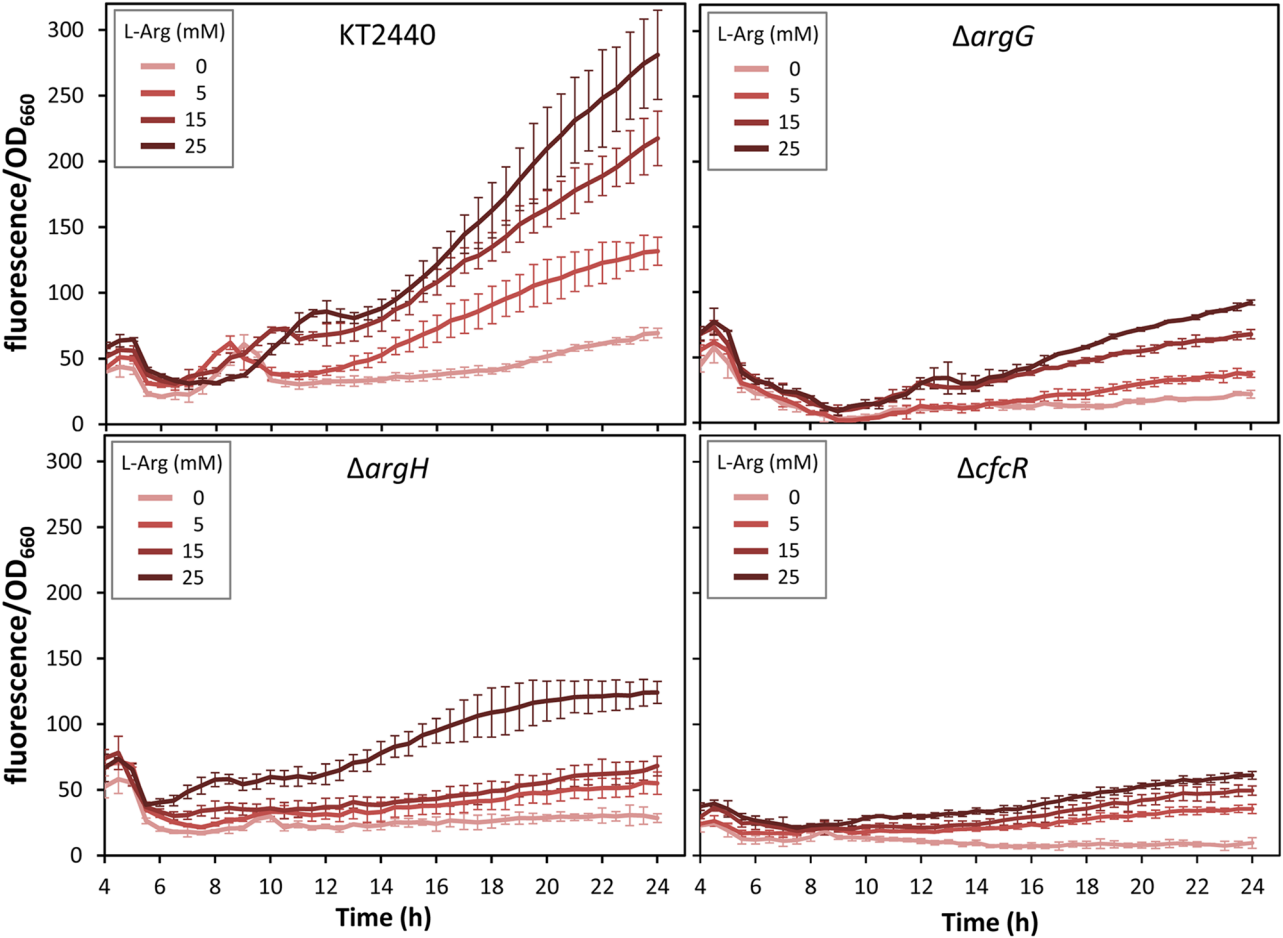
Modulation of c-di-GMP cell content by l-arginine in *P. putida* KT2440, the arginine biosynthesis mutants Δ*argG* and Δ*argH*, and the Δ*cfcR* mutant. Strains harbouring pCdrA::*gfp*^C^ were grown in diluted LB (1:3) supplied with different final concentrations of l-arginine (0, 5, 15 and 25 mM). Data correspond to the fluorescence values corrected by culture growth (OD_660_) over time. Averages and standard deviations of two biological replicates with three experimental replicates each are plotted. A Synergy Neo2 Biotek fluorimeter was used in these experiments.

To expand our previous observations connecting arginine biosynthesis and c-di-GMP levels, deletion mutants in *argG* and *argH*, previously constructed and confirmed to be auxotrophs for l-arginine^10^, were analysed in terms of second messenger contents by introducing the c-di-GMP biosensor plasmid pCdrA::*gfp*^C^^11^ and measuring fluorescence during growth in diluted LB. As observed earlier with transposon insertion mutants in these genes^7^, both the Δ*argG* and Δ*argH* strains showed significantly less fluorescence than the parental strain despite having similar growth patterns, indicative of reduced intracellular c-di-GMP contents (Supplementary Fig. S1), and lost the crinkly colony morphology associated to the plasmid harbouring *cfcR* in multicopy unless supplied with l-arginine (Supplementary Fig. S2). Addition of increasing concentrations of l-arginine to the growth medium strongly enhanced fluorescence in the wild type and restored it to a limited extent in the Δ*argG* and Δ*argH* mutants (Fig. 1). As expected, fluorescence was severely reduced in a Δ*cfcR* mutant even in the presence of l-arginine, although a certain dose-dependent response was still detectable (Fig. 1), suggesting that the raise of c-di-GMP levels due to the amino acid is mostly but not exclusively through the DGC activity of CfcR.

**Figure 2:**
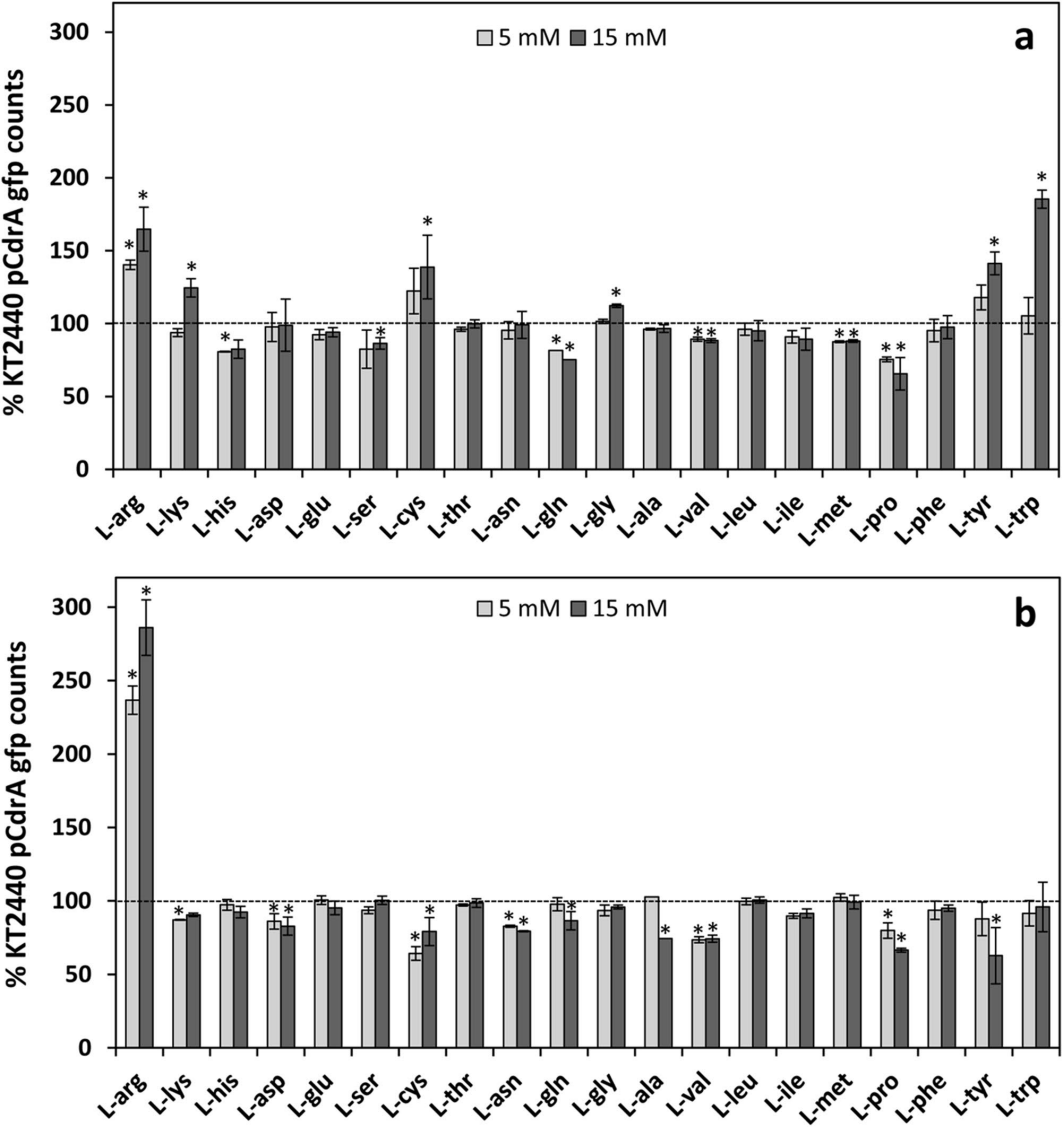
Modulation of c-di-GMP cell content by l-amino acids in *P. putida* KT2440. Cultures harbouring pCdrA*::gfp*^C^ were grown in 96-well plates during 24 h in 1:3 diluted LB **(a)** or M9 minimal medium with glucose **(b)** in the presence of each l-amino acid at 5 mM (light bars) or 15 mM (dark bars). Fluorescence and turbidity were quantified every 30 min for 24 h using a Tecan Infinite 200 fluorimeter. Values corresponding to the area under the curve derived from fluorescence measurements normalized by culture growth (OD_600_) were calculated, to obtain a global overview of fluorescence along the whole growth curve. Data are given as percentage relative to the value obtained for KT2440 (pCdrA*::gfp*^C^) without any added amino acid (established as 100%, dotted line). Averages and standard deviations of three independent experiments with three replicates each are presented. Values at least 10% higher or lower than the control and showing statistically significant differences with it are indicated by asterisks (Student´s *t* test; *p* ≤ 0.05).

To define if the stimulatory effect of l-arginine on c-di-GMP contents was specific of this amino acid, fluorescence of KT2440 harbouring pCdrA::*gfp*^C^ was tested during growth in rich and minimal medium supplied with each of the 20 proteinogenic l-amino acids at 5 or 15 mM during 24 h. As shown in Fig. 2, l-arginine was the only amino acid causing a relevant, concentration-dependent increase in relative fluorescence in both media (between 1.5- and threefold) throughout culture growth. Addition of 15 mM l-tryptophan also resulted in a relevant increase (nearly twofold) in rich medium but not in minimal medium, which could suggest the need for additional molecules for the response to l-tryptophan. Further analysis revealed a synergistic effect of l-arginine and l-tryptophan: addition of l-tryptophan in minimal medium had a minor influence on c-di-GMP levels, but the combination of both amino acids caused a significantly higher response than addition of l-arginine alone (Supplementary Fig. S3). Statistically significant, yet quantitatively less relevant increases were also observed with other l-amino acids. Negative effects could also be detected in some cases, particularly with proline, which caused a 35% reduction in relative fluorescence at 15 mM in both media (Fig. 2).

**Figure 3:**
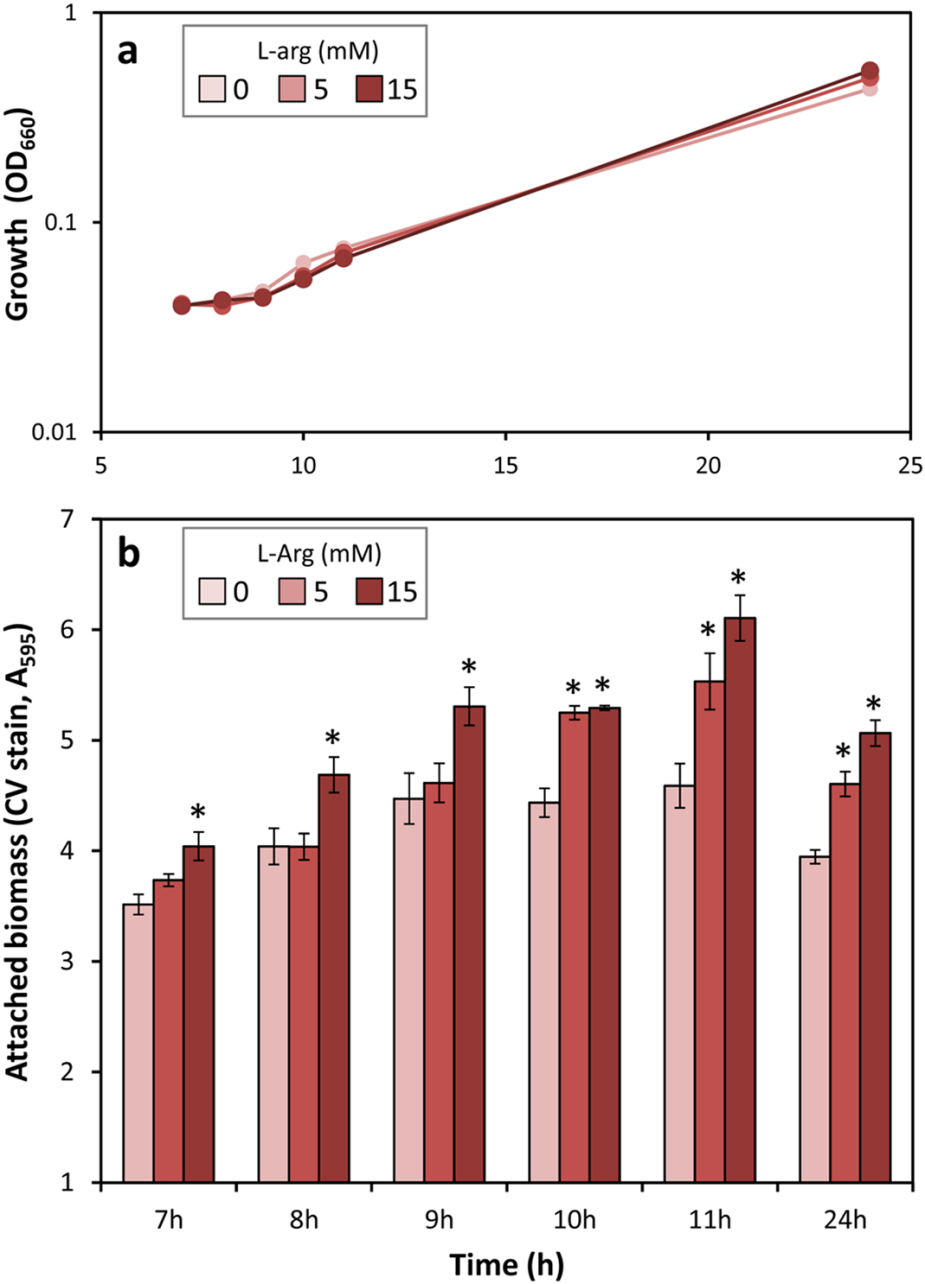
Effect of l-arginine on planktonic growth **(a)** and biofilm formation **(b)** by *P. putida* KT2440. Cultures were grown in 96-well plates in FAB medium with glucose and different concentrations of l-arginine (0, 5 and 15 mM). Growth was measured at 660 nm, and attached biomass was quantified as absorbance at 595 nm after staining with crystal violet (CV) and subsequent solubilisation of the dye. Results are averages and standard errors of two independent experiments with four technical replicates each. Asterisks indicate statistically significant differences with respect to the control without l-arginine (Student’s *t* test; *p* ≤ 0.05).

In many bacteria, including *P. putida* KT2440, c-di-GMP levels directly correlate with biofilm development. We therefore tested if increasing concentrations of l-arginine enhanced attachment and biofilm formation. Assays were done in polystyrene multiwell plates under static conditions in minimal medium with glucose as carbon source. The presence of l-arginine did not influence planktonic growth in these conditions (Fig. 3a), but increased the amount of attached biomass (Fig. 3b).

### Arginine biosynthesis modulates the expression of biofilm structural elements

**Figure 4:**
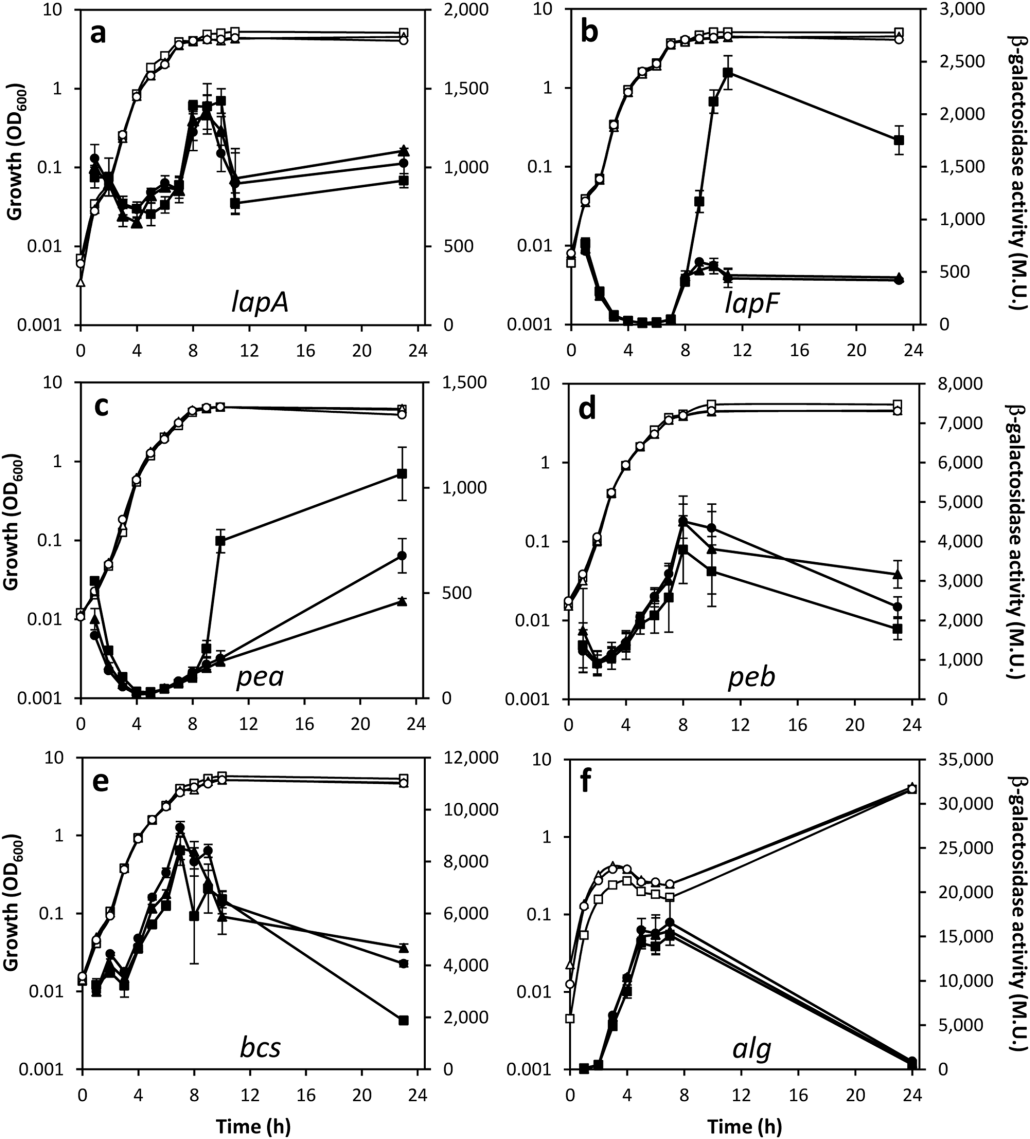
Influence of Δ*argG* and Δ*argH* deletions on expression of adhesin- and EPS-encoding genes. KT2440 (circles), Δ*argG* (triangles) and Δ*argH* (squares) strains carrying reporter fusions corresponding to *lapA::lacZ* (**a**), *lapF::lacZ* (**b**), *pea* (*PP_3132::lacZ*) (**c**), *peb* (*PP_1795::lacZ*) (**d**), *bcs* (*PP_2629::lacZ*) (**e**), and *alg* (*algD::lacZ*) (**f**) were grown in LB. Turbidity (OD_600_, hollow symbols) and β-galactosidase activity (Miller units, solid symbols) at the indicated time points are shown. d-cycloserine (75 μg/ml) was added in **f** after 1 h of growth, since the *algD* promoter is inactive in the absence of cell wall stress in *P. putida*^16^. The data are averages and standard deviations of at least two biological replicates with two technical repetitions each.

The crinkly colony phenotype associated to high levels of c-di-GMP in *P. putida* requires the species-specific exopolysaccharide (EPS) Pea^5^. On the other hand, the large adhesins LapA and LapF are essential for the development of mature biofilms in *P. putida*, a process in which the four EPS present in this bacterium would contribute differently depending on the environmental conditions^12–14^. These elements are differentially modulated by the c-di-GMP dependent regulator FleQ^14–16^. All these facts prompted us to investigate if expression of any of those structural elements of the biofilm matrix was affected in the Δ*argG* and Δ*argH* mutants. Plasmids harbouring transcriptional fusions of *lapA*, *lapF* and the first gene in each EPS cluster with the reporter gene *lacZ* devoid of its own promoter^16–18^ were introduced in *P. putida* KT2440 and the two mutants and β-galactosidase activity was followed during growth in LB. Results are summarized in Fig. 4. A significant reduction in expression was observed in stationary phase for the *lapF::lacZ* and the *pea::lacZ* fusions (Fig. 4b,c) in both arginine biosynthesis mutants compared to the wild type. In contrast, the other four fusions showed only minor differences between strains.

**Figure 5:**
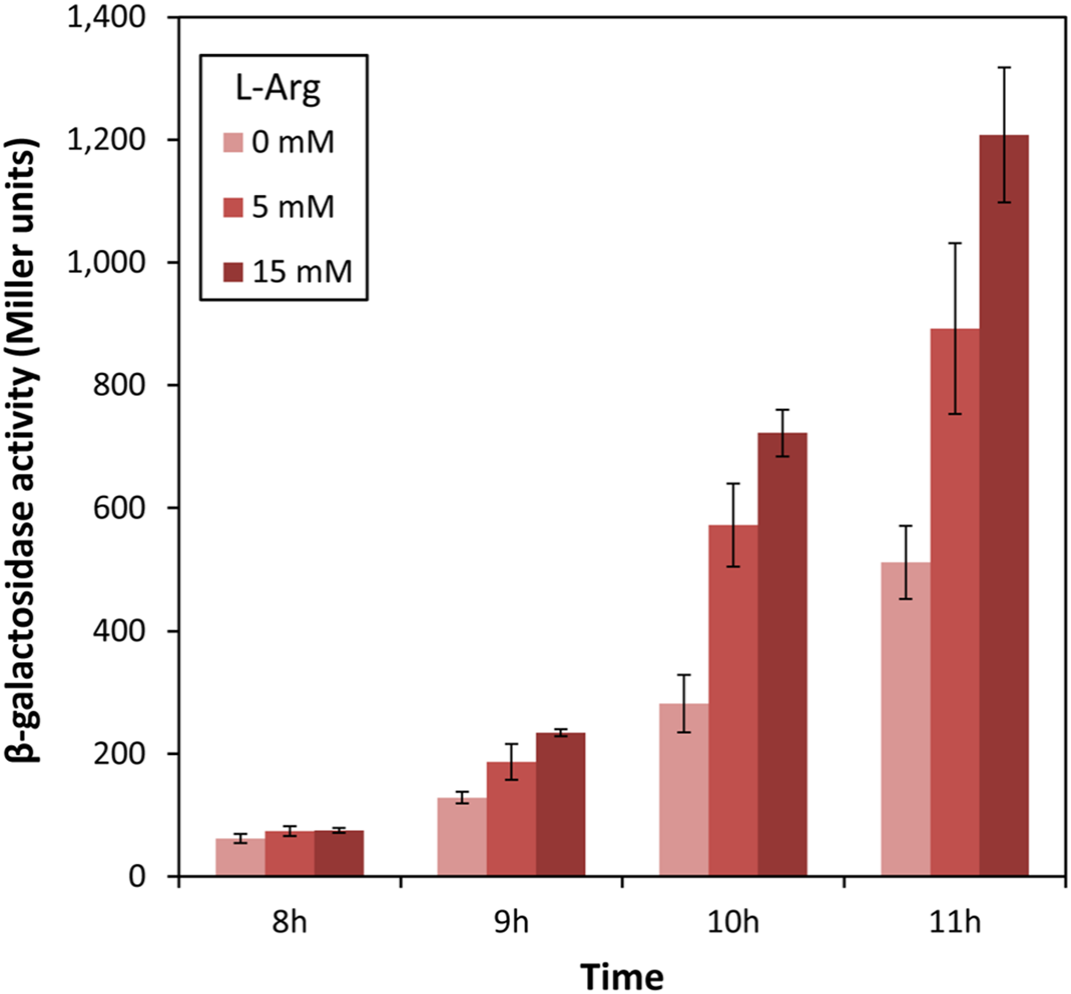
l-arginine increases expression of *pea*. KT2440 harbouring the *PP_3132::lacZ* fusion was grown in M9 minimal medium with glucose as carbon source, supplied with 0, 5, and 15 mM l-arginine (shown as increasing intensity colour bars), and β-galactosidase activity was measured at different time points. The experiment was done in duplicate with three technical repetitions each. Statistically significant differences were observed at 8, 9, 10 and 11 h between the absence and presence of l-arginine (Student´s *t* test; *p* ≤ 0.05), but quantitatively relevant differences were obvious only at 10 and 11 h (early stationary phase).

Interestingly, the appearance of the crinkly colony phenotype associated to high levels of c-di-GMP does not take place in *P. putida* KT2440 harbouring *cfcR* in multicopy when grown on M9 minimal medium agar plates with glucose as the only carbon source, unless l-arginine is added (Supplementary Fig. S2). We therefore tested if expression of *pea*, required for this phenotype, also responded to exogenous l-arginine. As shown in Fig. 5, expression of the *pea::lacZ* fusion was enhanced with increasing concentrations of the amino acid. This effect was more evident upon entry in stationary phase.

### Expression of *rpoS* is influenced by exogenous and endogenous l-arginine

**Figure 6:**
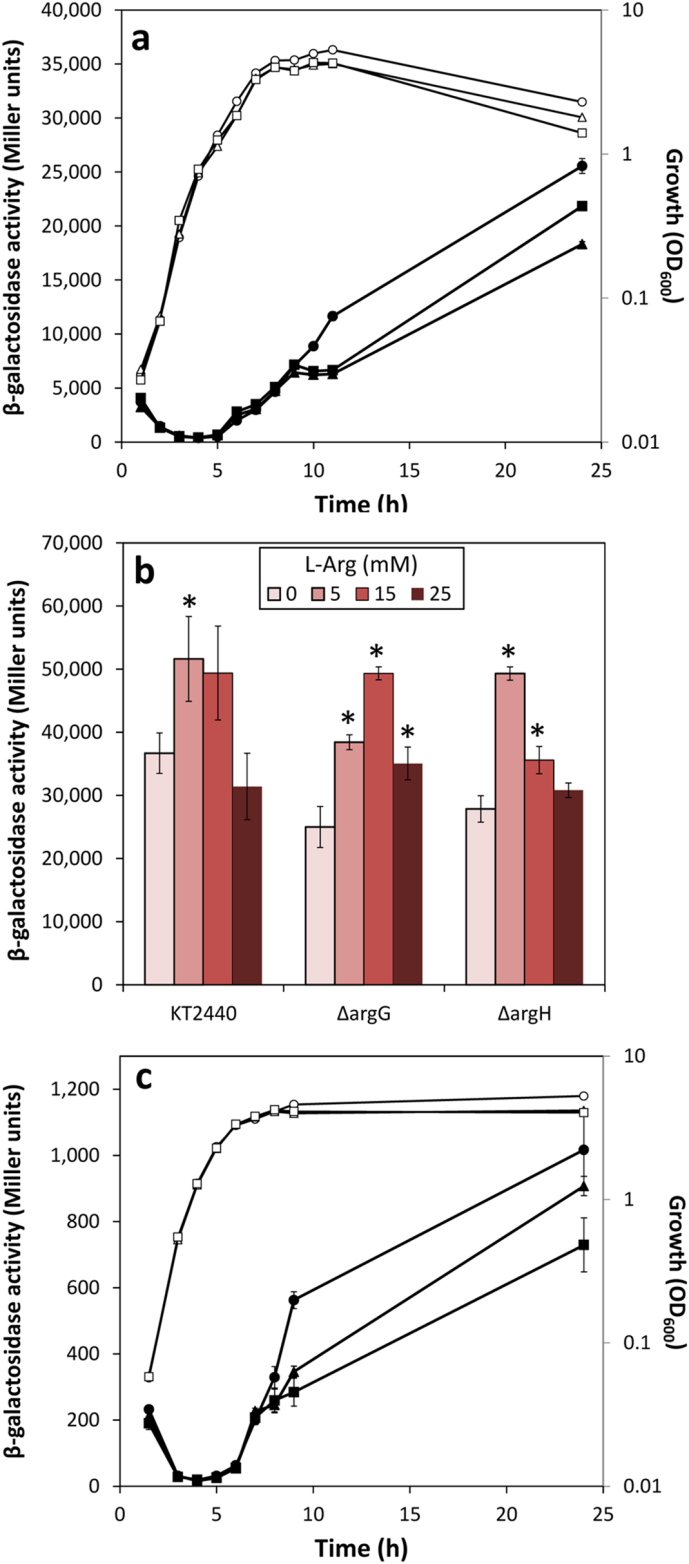
Influence of l-arginine biosynthesis on expression of *rpoS* and *cfcR*. **(a)** KT2440 (circles), and the Δ*argG* (triangles) and Δ*argH* (squares) strains harbouring pMAMV21 (*rpoS’–‘lacZ*) were grown in LB and β-galactosidase activity was measured at the indicated times. Data correspond to averages and standard errors of two biological replicas with three technical repetitions each. Statistically significant differences between the wild type and mutants were detected from 10 h onwards (Student´s *t* test: *p* < 0.05). **(b)** Influence of increasing concentrations of l-arginine on expression of *rpoS’–‘lacZ* in KT2440 and the Δ*argG* and Δ*argH* strains harbouring pMAMV21. Cultures were grown for 24 h in LB or LB with increasing concentrations of l-arginine (shown as increasing intensity colour bars) and β-galactosidase activity was analysed. Graph corresponds to averages and standard deviations of two biological replicas with three technical repetitions each. Statistically significant differences with respect to each control without amino acid supplementation are indicated by asterisks (Student´s *t* test: *p* ≤ 0.01). **(c)** KT2440 (circles), and the Δ*argG* (triangles) and Δ*argH* (squares) strains harbouring pMIR200 (*cfcR::lacZ*) were grown in LB and β-galactosidase activity was measured at the indicated times. Data correspond to averages and standard errors of two biological replicas with three technical repetitions each.

Expression of *cfcR* and *lapF* is under the control of the stationary phase sigma factor RpoS^5,17^, and the same has been recently reported for *pea*^19^, a result that we have independently confirmed (Supplementary Fig. S4). Hence, we considered the possibility that the differences in expression observed for these two genes in the arginine biosynthesis mutants could reflect an influence of arginine availability on expression of *rpoS*. To test this hypothesis, a translational *rpoS’–‘lacZ* fusion, harboured in pMAMV21^5^, was introduced in *P. putida* KT2440 and the Δ*argG* and Δ*argH* mutants, and β-galactosidase activity was measured during growth in LB. Results in Fig. 6a, indicate that a functional arginine biosynthesis pathway is required for full expression of *rpoS* expression, since β-galactosidase activity was reduced in both mutants in stationary phase. Addition 5 or 15 mM of l-arginine increased β-galactosidase activity in the Δ*argG* and Δ*argH* mutants (Fig. 6b), whereas addition of 25 mM of the amino acid caused a less stimulatory effect compared with 15 mM in the case of the Δ*argG* mutant and had no significant effect in the Δ*argH* strain. In the wild type, significantly increased *rpoS* expression was only observed with 5 mM l-arginine. Activity of a transcriptional *cfcR::lacZ* fusion, harboured in pMIR200 ^6^, was also tested in the Δ*argG* and Δ*argH* mutants. As shown in Fig. 6c, the expression pattern of *cfcR* was similar to that observed for *rpoS*, with the mutants having lower activity upon entry into stationary phase.

### Substrate binding proteins participate in the response to environmental l-arginine

To explore in more detail the response of *P. putida* to l-arginine, we carried out a similarity search analysis to identify proteins that could be analogous to ArtI, the l-arginine binding protein involved in c-di-GMP signalling in *S. enterica* serovar Typhimurium ^9^. Two proteins, corresponding to loci PP_0282 and PP_4486, present around 40% identical residues with ArtI of *S. enterica*, and a third one, encoded by PP_3593, shows 36% identity. The three are periplasmic substrate binding proteins sharing around 25% identical residues, amino acids likely involved in arginine binding are conserved, and the corresponding genes are located in clusters encoding predicted amino acid ABC transporters (Supplementary Fig. S5). PP_0282 is annotated in the *Pseudomonas* genome database (https://www.pseudomonas.com; ^20^) as ArtJ (l-arginine ABC transporter substrate-binding subunit) and PP_4486 as ArgT (lysine/arginine/ornithine ABC transporter substrate-binding protein); PP_3593 has no specific annotation, but the protein is 72% identical to the octopine-binding protein OccT of *Pseudomonas protegens* CHA0. Hereafter, this nomenclature is followed.

**Figure 7:**
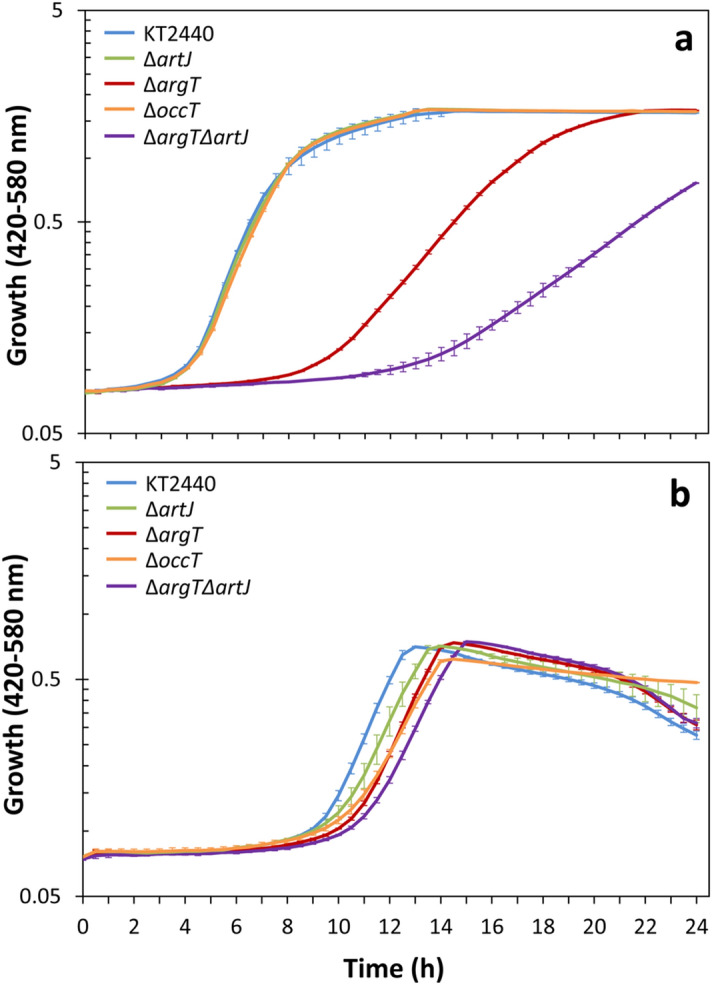
Growth of *P. putida* KT2440 and mutant derivatives with l-arginine as nitrogen (**a**) or carbon and energy source (**b**). Strains KT2440 (blue lines), Δ*argT* (crimson lines), Δ*artJ* (green lines), Δ*occT* (orange lines), and Δ*argT*Δ*artJ* (purple lines) were grown in a Bioscreen C MBR apparatus at 30 °C with shaking during 24 h in 100-well plates in M8-glucose or M9 minimal medium with 10 mM l-arginine. Absorbance in the 420–580 nm range was measured every 30 min. Three independent assays were done with three technical replicas each. Averages and standard deviations of one representative experiment are shown.

To define the potential role of these substrate-binding proteins in l-arginine transport, deletion mutants were constructed in each of the corresponding genes, as well as a double Δ*argT*Δ*artJ* mutant, and their growth was tested in M8 minimal medium with glucose as cabon source and l-arginine as nitrogen source. Results presented in Fig. 7a indicate that ArgT is the main contributor to l-arginine uptake, given the long lag phase and extended doubling time of the Δ*argT* mutant. The Δ*occT* and Δ*artJ* mutants were not affected in growth, whereas the double Δ*argT*Δ*artJ* mutation caused a much stronger effect on growth than the single Δ*argT* mutation, suggesting that in the absence of ArgT, ArtJ also plays a relevant role in l-arginine uptake. When l-arginine was supplied as the sole carbon and energy source in M9 minimal medium, all mutants showed a slight delay in growth, being greater in the double mutant (Fig. 7b). Experiments with other basic amino acids as nitrogen or carbon sources revealed only minor differences between strains (Supplementary Fig. S6), except in the case of the Δ*occT* mutant, which was unable to grow in l-lysine as carbon and energy source.

**Figure 8:**
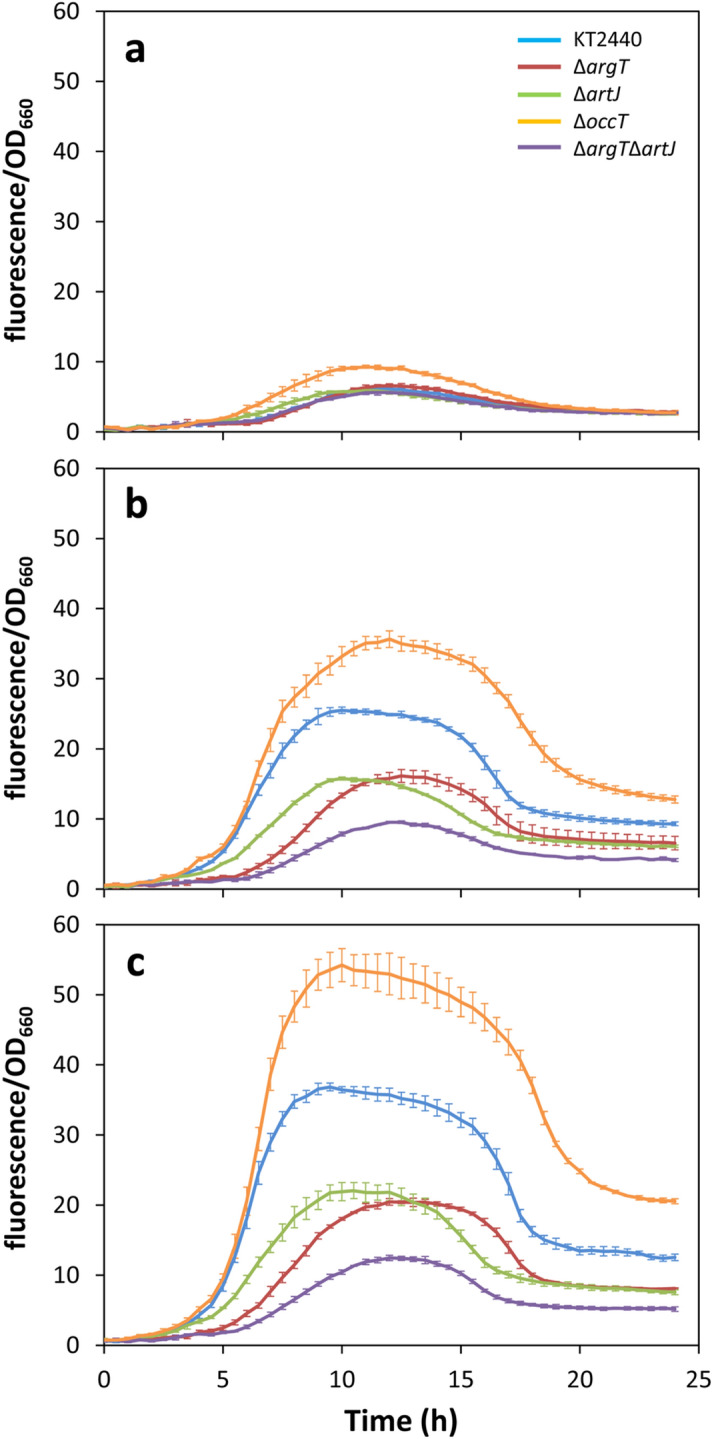
Role of substrate binding proteins in modulation of c-di-GMP cell content by environmental l-arginine. *P. putida* KT2440 (blue lines), Δ*argT* (crimson lines), Δ*artJ* (green lines), Δ*occT* (orange lines), and Δ*argT*Δ*artJ* (purple lines) strains harbouring pCdrA*::gfp*^C^ were grown in M9 minimal medium with glucose (**a**) and 5 mM (**b**) or 15 mM (**c**) l-arginine. Data correspond to fluorescence values corrected by culture growth (OD_660_). Measurements were done every 30 min on a Varioskan Lux fluorimeter. Averages and standard deviations are plotted.

To test the involvement of these binding proteins in arginine-dependent c-di-GMP signalling, the biosensor pCdrA*::gfp*^C^ was introduced in the Δ*argT*, Δ*artJ*, Δ*occT* and Δ*argT*Δ*artJ* mutants, and fluorescence was analysed during growth in M9 minimal medium with glucose as carbon source and in the presence of increasing concentrations of l-arginine. As shown in Fig. 8, no difference in relative fluorescence was observed between the wild type and the Δ*argT*, Δ*artJ*, and Δ*argT*Δ*artJ* mutants in the absence of l-arginine (Fig. 8a). However, the dose-dependent response to l-arginine was significantly reduced in all these mutants, a cumulative effect being observed in the double Δ*argT*Δ*artJ* mutant in these conditions (Fig. 8b,c)*.* Surprisingly, the Δ*occT* mutant showed increased fluorescence with respect to KT2440 in the absence of the amino acid (Fig. 8a) and maintained the dose-dependent response to l-arginine, reaching higher fluorescence levels than the wild type at the different concentrations of amino acid tested (Fig. 8b,c).

### Role of arginine binding proteins in biofilm development associated to l-arginine

**Figure 9:**
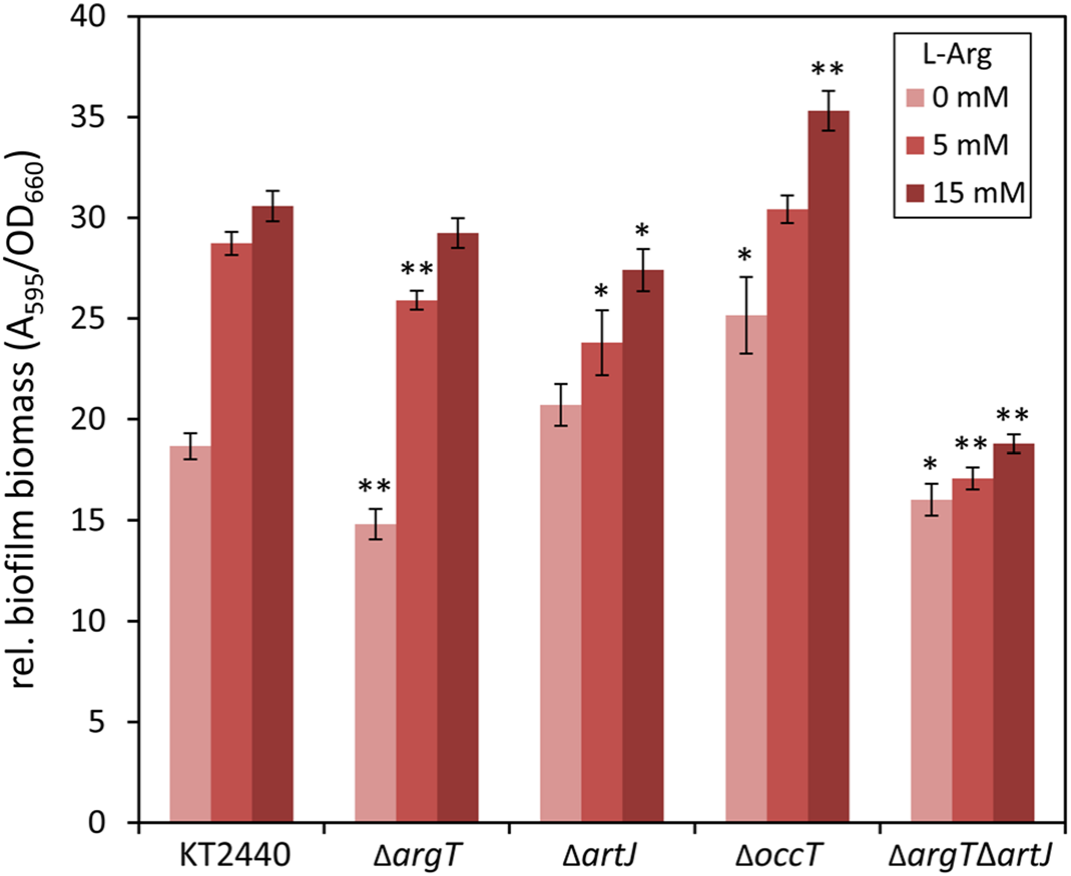
Influence of periplasmic substrate binding proteins on biofilm formation by *P. putida*. KT2440 and the Δ*argT*, Δ*artJ*, Δ*argT*Δ*artJ*, and Δ*occT* mutants were grown in FAB minimal medium with glucose and different l-arginine concentrations (shown as increasing intensity colour bars). Attached biomass was analysed after 10 h of growth. Values correspond to absorbance (A_595_) after staining with crystal violet and subsequent solubilisation of the dye, normalized with respect to culture growth (OD_660_). Data are averages and standard errors from two independent experiments with four technical replicates each. Asterisks indicate statistically significant differences between the wild type and the corresponding mutant in each condition (Student’s *t* test; *p ≤ 0.05; **p ≤ 0.01).

We tested if the above results correlated with changes in biofilm formation between the wild type and the different mutants in the presence or absence of exogenous l-arginine. Results are shown in Fig. 9. As previously observed, addition of the amino acid resulted in an increase in biofilm formation in KT2440, an effect that was reduced in the Δ*artJ* mutant and almost completely abolished in the double Δ*argT*Δ*artJ* mutant. The Δ*argT* mutant, on the other hand, showed a reduction in biofilm biomass in all the conditions tested compared to the wild type (although not statistically significant when grown with 15 mM l-arginine), but retained a dose-dependent response to the amino acid. In contrast, the Δ*occT* mutation caused the opposite effect, consistently with the increase in c-di-GMP observed in this mutant.

## Discussion

In recent years, evidence has been accumulating that connects bacterial social behaviours with the presence in the environment of certain amino acids. d-amino acids prevent biofilm formation in *Staphylococcus aureus* and *P. aeruginosa*^21^. On the other hand, several l-amino acids have been described to hamper swarming motility and stimulate biofilm formation in *P. aeruginosa* PA14^8^; among them, arginine caused a significant increase in c-di-GMP content, even though it was not the most relevant in terms of enhancing biofilm formation^8^. However, the positive effect of arginine on biofilm formation was only observed in cultures grown with the amino acid as the only carbon and nitrogen source, but not when both arginine and glucose were present^8^. In contrast, our results show that l-arginine increases c-di-GMP levels and promotes biofilm formation in *P. putida* regardless of the presence of other carbon and nitrogen sources. Furthermore, in this bacterium l-arginine appears to function both as a metabolic signal and as an environmental signal: mutants deficient in arginine biosynthesis show low c-di-GMP levels, partly restored by exogenous l-arginine, while mutants limited in arginine transport present reduced response to the presence of the amino acid in the growth medium, in terms of c-di-GMP levels and biofilm formation. We have also confirmed previous observations on the importance of l-arginine in the development of crinkly colony morphology^7^, a phenotype associated to high levels of c-di-GMP in *P. putida* KT2440 which requires of the species-specific EPS Pea^4^. Addition of l-arginine is required for this phenotype to develop in minimal medium, and the amino acid specifically restores this phenotype in mutants deficient in arginine biosynthesis, indicating that l-arginine plays a relevant role in Pea production. Accordingly, expression of Pea is significantly reduced in Δ*argG* and Δ*argH* mutants and increased by addition of l-arginine to the growth medium. EPS production dependent on the presence of l-asparagine in the culture medium has been reported in *Bacillus*^22^*.* Interestingly, amino acid-decorated EPSs have been identified in *Vibrio*^23,24^. Whether arginine is a component of the EPS Pea in *P. putida* remains unknown.

The effect of exogenous l-arginine on motility and biofilm development can vary in different bacteria depending on the concentration of amino acid. For instance, in *Streptococcus gordonii*, low l-arginine concentrations (between 0.5 and 500 µM) enhance biofilm development and promote the establishment of structured biofilms, while high concentrations (≥ 50 mM) alter biofilm architecture, biomass and thickness^25^. In the case of *P. aeruginosa* PAO1, l-arginine concentrations above 250 mM inhibit swimming motility, whereas lower concentrations (100 mM) favour this type of motility ^26^. In this work we have observed positive effects on biofilm formation and c-di-GMP levels with l-arginine concentrations ranging between 5 and 25 mM, but we have also seen different responses in terms of *rpoS* expression depending on l-arginine concentration. Other l-amino acids also seem to have a positive or negative influence on c-di-GMP levels in KT2440, but to a lesser and in some cases variable extent depending on the growth medium. Among them, l-tryptophan causes a significant increase in c-di-GMP levels in rich medium but not in minimal medium, and our data indicate the existence of a synergistic effect of l-arginine and l-tryptophan. This amino acid has been described to positively impact biofilm development in *S. enterica* serovar Typhimurium^27^, and genes related to tryptophan biosynthesis are upregulated during early biofilm formation in *E. coli*^28,29^. However, no effect of tryptophan alone was reported in *P. aeruginosa* PA14^8^. The connection between arginine and tryptophan signalling will deserve further detailed exploration.

In these experiments, the negative effect of l-aspartic acid, previously shown to reduce c-di-GMP levels in KT2440^7^, was not so evident. It should be noted that the data presented in Fig. 2 correspond to the area below the curve for the relative fluorescence data over 24 h, in order to assess overall differences. Changes in c-di-GMP levels with l-aspartic acid were only evident at late times of growth^7^, and are therefore likely underscored when data throughout 24 h of bacterial culture are compiled together. The negative effect of l-aspartic acid was further evidenced by the reduction of the crinkle colony morphology of KT2440 harbouring *cfcR* in multicopy when grown in the presence of increasing concentrations of the amino acid (Supplementary Fig. S2). It could also explain why arginine supplementation did not fully restore c-di-GMP levels in the Δ*argG* and Δ*argH* mutants (Fig. 1), since these mutants are bound to accumulate aspartic acid^7^.

There is still limited information about the mechanisms of action of amino acids that lead to changes in the turnover of the second messenger. Our results indicate that in *P. putida* KT2440, the response regulator with DGC activity CfcR, the chief contributor to c-di-GMP levels in stationary phase^6^, is the main element in the increase in c-di-GMP levels caused by exogenous l-arginine, despite its lack of amino acid-binding or protein–protein interaction domains. Still, a Δ*cfcR* mutant retains some response to l-arginine, suggesting additional protein(s) with DGC activity yet to be identified also participate in the process. In *P. aeruginosa* PAO1, SadC and RoeA, two of the most important DGCs implicated in biofilm formation, are necessary for the l-arginine response ^8^. In addition, a multidomain transmembrane protein with PDE activity encoded by locus PA0575 (RmcA) binds l-arginine in its N-terminal domain, and a mutant in this gene shows increased c-di-GMP levels in response to the amino acid ^30^. Homologs of SadC or RoeA are missing in *P. putida* KT2440, but a homolog of RmcA can be found (PP_0386). It will be worth exploring its potential contribution to the arginine response, although it would be expected to correlate with a decrease rather than an increase in the levels of c-di-GMP, based on its role in *P. aeruginosa*.

Arginine has also been found to induce the synthesis of c-di-GMP in *Salmonella enterica* serovar Thyphimurium. Although the mechanism is not fully characterized, the substrate binding subunit ArtI of the arginine transporter and the diguanylate cyclase STM1987, containing a periplasmic sensing domain, are required for the response to the amino acid ^9^. *P. putida* KT2440 does not appear to have an equivalent of STM1987, but our results show that substrate binding proteins associated to amino acid transport systems are important for the response to l-arginine: mutants lacking ArgT and/or ArtJ, both of which participate in arginine transport, partially lose the increase in c-di-GMP levels observed in *P. putida* KT2440 in the presence of the amino acid. In contrast, deletion of a third substrate binding protein, OccT, limits lysine utilization as carbon source (Supplementary Fig. S6) but has little influence on arginine transport and causes an increase in c-di-GMP levels. The transport systems associated to ArgT and ArtJ had been previously described to participate in l-lysine transport^31^. It has been reported that in KT2440, two active metabolic pathways are required for utilization of l-lysine as the sole carbon source: the aminovalerate pathway and the aminoadipate pathway^32^. The second one requires conversion of l-lysine to d-lysine by a periplasmic racemase^33^. Since the *occT* mutant can use l-lysine as nitrogen source but not as carbon source, it seems plausible that this transport system is in fact required for d-lysine uptake, and that d-lysine, as described for other d-amino acids in different bacteria, reduces c-di-GMP levels in *P. putida*. This would be consistent with the increased second messenger levels detected in the *occT* mutant. Such idea is further supported by the fact that the *occT* gene is in the same genomic context as genes related to d-lysine catabolism ^33^, but additional work will be required to confirm it.

We hypothesize all these data, along with those obtained with arginine biosynthesis mutants, indicate that cellular arginine pools are sensed and transduced into c-di-GMP turnover and signalling in *P. putida*. This notion, rather than direct interaction between a substrate binding protein and a DGC, is compatible with the changes in expression of RpoS and elements under its control (*lapF*, *pea*, *cfcR*) in the Δ*argG* and Δ*argH* mutants, and the influence of exogenous l-arginine through periplasmic binding proteins associated to different amino acid transport systems. It is possible that two independent signalling pathways exist for intracellular and extracellular arginine. Yet, if our hypothesis is correct, the results presented here open the way to further exploring a still poorly developed area of research, namely how central metabolism and second messenger turnover are connected in bacteria. The underlying molecular mechanisms will be analysed in future work.

## Methods

### Bacterial strains, culture media and growth conditions

Strains used in this work are listed in Table 1. *Pseudomonas putida* KT2440 is a plasmid-free derivative of *P. putida* mt-2, which was isolated from a vegetable orchard in Japan and whose genome is completely sequenced^34,35^. *Pseudomonas* strains were routinely grown at 30 °C in Luria–Bertani (LB) medium^36^. Where indicated, M9^37^ or modified FAB^38^ minimal media supplied with glucose (20 mM) as carbon source were used. *Escherichia coli* strains were grown at 37 °C in LB. When appropriate, antibiotics were used at the following concentrations (μg/ml): chloramphenicol (Cm) 30; kanamycin (Km) 25; tetracycline (Tc) 10; gentamicin (Gm) 10 (for *E. coli*) or 100 (for *P. putida*), piperacillin (Pip) 30; ampicillin (Ap) 100; streptomycin (Sm) 50 (for *E. coli*) or 100 (for *P. putida*).

**Table 1:** Bacterial strains and plasmids used.

Strain or plasmid	Genotype/relevant characteristics	Reference or source
**Strains**		
*E. coli*		
CC118λpir	Rif^R^, λpir	^47^
DH5α	*supE44 lac*U169 (Ø80*lacZΔ*M15) *hsd*R17 (r_K_-m_k_-) *recA*1 *endA*1 *gyrA*96 *thi*-1 *rel*A1	^48^
HB101 (pRK600)	Helper strain harbouring Cm^R^*mob tra* plasmid	^47^
*P. putida*		
KT2440	Wild type; derivative of *P. putida* mt-2, cured of pWWO	PRCC^a^
Δ*argG*	Null mutant derivative of KT2440 in PP_1088 (*argG*)	^10^
Δ*argH*	Null mutant derivative of KT2440 in PP_0184 (*argH*)	^10^
Δ*argT*	Null mutant derivative of KT2440 in PP_4486 (*argT*)	This work
Δ*artJ*	Null mutant derivative of KT2440 in PP_0282 (*artJ*)	This work
Δ*occT*	Null mutant derivative of KT2440 in PP_3593 (*occT*)	This work
Δ*argT*Δ*artJ*	Double null mutant derivative of KT2440 in PP_4486 (*argT*) and PP_0282 (*artJ*)	This work
Δ*cfcR*	Null mutant derivative of KT2440 in PP_4959 (*cfcR*)	^5^
**Plasmids**		
pCR2.1 TOPO	Km^R^, cloning vector with β-galactosidase α-complementation	Invitrogen
pCdrA::*gfp*^*C*^	Ap^R^ (Pip^R^), Gm^R^, FleQ dependent c-di-GMP biosensor	^11^
pKNG101	Sm^R^, *oriR6K mobRK2 sacBR*	^42^
pLBM30	pCR2.1TOPO derivative with 1.449 Kb *NotI* fragment containing the *argT* null allele	This work
pLBM31	pCR2.1TOPO derivative with 1.358 Kb *NotI* fragment containing the *artJ* null allele	This work
pLBM32	pCR2.1TOPO derivative with 1.4 Kb *NotI* fragment containing the *occT* null allele	This work
pLBM33	Sm^R^, pKNG101 derivative for *argT* null allele replacement with the 1.449 Kb *NotI* fragment of pLBM30 cloned at the same site of pKNG101	This work
pLBM34	Sm^R^, pKNG101 derivative for *artJ* null allele replacement with the 1.358 Kb *NotI* fragment of pLBM31 cloned at the same site of pKNG101	This work
pLBM35	Sm^R^, pKNG101 derivative for *occT* null allele replacement with the 1.4 Kb *NotI* fragment of pLBM32 cloned at the same site of pKNG101	This work
pMIR125	Tc^R^; transcriptional fusion *algD:: lacZ* containing RBS and first codons in pMP220	^16^
pMP220-*bcs*	Tc^R^; transcriptional fusion PP_2629*::lacZ* containing RBS and first codons in pMP220	^16^
pMP220-*pea*	Tc^R^; transcriptional fusion PP_3132*::lacZ* containing RBS and first codons in pMP220	^16^
pMP220-*peb*	Tc^R^; transcriptional fusion PP_1795*::lacZ* containing RBS and first codons in pMP220	^16^
pMMG1	Tc^R^; transcriptional fusion *lapF::lacZ* containing RBS and first codons in pMP220	^17^
pMMGA	Tc^R^; transcriptional fusion *lapA::lacZ* containing RBS and first codons in pMP220	^18^
pMAMV21	Tc^R^, translational fusion *rpoS’–‘lacZ* in pMP220-*BamHI*	^5^
pMIR200	Tc^R^, transcriptional fusion *cfcR::lacZ* in pMP220	^6^

*Rif* rifampin, *Cm* chloramphenicol, *Km* kanamycin, *Tc* tetracycline, *Sm* streptomycin, *Pip* piperacillin, *Ap* ampicillin, *Gm* gentamicin.

^a^*Pseudomonas* Reference Culture Collection (https://artemisa.eez.csic.es/prcc/).

### Molecular biology techniques

DNA preparation, digestion with restriction enzymes, plasmid dephosphorylation, adenylation, ligation and cell transformations were carried out using standard protocols^39,40^. PCR amplifications were done using Phusion High-Fidelity DNA polymerase (Thermo Fisher Scientific). Plasmid purification and gel extraction from agarose gels were done with appropriate kits, following manufacturers´ instructions (NZYTech and QIAgen, respectively). Transfer of plasmids to *Pseudomonas* cells was performed by electrotransformation or triparental conjugation as previously described^10,41^.

### Construction of null mutants

Null mutants were obtained by gene replacement of the wild type allele with a null allele via homologous recombination, without inserting any antibiotic resistance marker. The strategy designed to obtain the mutants consisted of the amplification of the upstream and downstream fragments surrounding the gene to be replaced by overlapping PCR using Phusion High-Fidelity DNA polymerase (Thermo Fisher Scientific). Oligonucleotides used are detailed in Supplementary Table S1. PCR reactions were carried out in two steps. Firstly, flanking regions of the gene to be removed were amplified separately using primers with *Not*I restriction site on one end and a complementary tail on the other end. Secondly, overlapping upstream and downstream regions were used as template for the second PCR, obtaining a single amplicon flanked with *Not*I restriction sites. PCR product was cloned into pCR2.1-TOPO vector after its adenylation, transferred to *E. coli* DH5α by heat shock transformation, and sequenced to ensure the absence of mutations. The fragment was then subcloned into the *Not*I site of the suicide vector pKNG101, which is unable to replicate in *Pseudomonas* and allows the generation and selection of double recombination events^42^. Each pKNG101 derivative containing the mutation was mobilized from *E. coli* CC118λ*pir* to *P. putida* KT2440 by triparental conjugation^10^. Merodiploid exconjugants were selected in M9 minimal medium with citrate as carbon source and streptomycin. One of them was selected to obtain clones in which a double recombination event had taken place after growth in LB medium supplied with 14% sucrose. Resulting mutants were sucrose-resistant and streptomycin-sensitive. Null mutants were checked by PCR, followed by sequencing of the corresponding genome region.

### Growth curves

To analyse the growth of *P. putida* KT2440 and its mutant derivatives in basic l-amino acid transport, overnight cultures grown on glucose-M9-plates at 30 °C were scrapped out in 1 ml of M9 salts and washed two times in the same medium. Inocula were adjusted to a final optical density at 660 nm (OD_660_) of 0.02 in M9 salts and distributed in 100-well plates (150 µL/well). l-arginine, l-lysine, l-histidine or l-ornithine were added as carbon and energy sources at a final concentration of 10 mM. Alternatively, M8 minimal medium with glucose as carbon and energy source and the amino acids supplied as nitrogen source, was used. Plates were incubated at 30 °C with continuous shaking (200 r.p.m.) and growth of the cultures was monitored at 30 min intervals for 24 h in an automated BioScreen C MBR apparatus equipped with a wide band filter (420–580 nm).

### Biofilm assays

Biofilm formation assays were performed in 96-well polystyrene microtiter plates as previously described^43^, using modified FAB medium with glucose as carbon source, based on the presence of calcium in its composition, which is important for adhesion^44,45^. Briefly, overnight cultures were diluted to an OD_660_ of 0.02 and 150 µL were added to each well. Where indicated, l-arginine was added at final concentrations of 5 or 15 mM. Plates were incubated at 30 °C in static conditions. At the indicated times, growth of the cultures (OD_660_) was measured, liquid was removed and wells were washed twice with distilled water. Biomass attached to the surface was stained with crystal violet (0.4%) for 15 min and quantified after dye solubilisation with glacial acetic acid (30% v/v) by measuring absorbance at 595 nm in a Tecan Sunrise plate reader.

### Measurement of β-galactosidase activity

β-galactosidase activity was assayed during growth in LB as described^46^. Alternatively, where indicated, M9 minimal medium with glucose, with or without l-arginine was used. Overnight cultures were diluted to an optical density of 0.05 in fresh medium. After 1 h of growth at 30 °C and 200 rpm, cultures were diluted 1:10 to ensure proper dilution of β-galactosidase that might have accumulated after overnight growth; this step was omitted when experiments were done in minimal medium. Incubation was continued in the same conditions, collecting samples at the indicated times. The results are expressed in Miller units and correspond to averages and standard deviations of at least two independent experiments with three technical replicas per sample.

### Comparative analysis of c-di-GMP levels based on a bioreporter

The bioreporter plasmid pCdrA::*gfp*^C^ was used for quantitative analysis of c-di-GMP levels based on fluorescence. This plasmid carries a fusion of *gfp* to the promoter of the *P. aeruginosa* gene *cdrA*, which responds to c-di-GMP via the transcriptional regulator FleQ^11^. Overnight cultures were diluted in fresh medium (LB diluted 1:3 or M9 with glucose) to a final OD_600_ of 0.02 and distributed into suitable 96-well plates (Greiner or Nunc Flat Bottom Black Polystyrol 96-well plates). Where indicated, l-amino acids were added at final concentrations of 5, 15 or 25 mM. Plates were incubated at 30 °C in static conditions and growth (OD_660_) and fluorescence (excitation: 485 nm, emission: 535 nm) were monitored every 30 min for 24 h using microplate fluorescence readers equipped with shaking and temperature control (TECAN Infinite 200, Synergy Neo2 Biotek, and Varioskan Lux). Data are presented as fluorescence/OD_600_. In the case of Fig. 2, data correspond to the calculated area below the curve for all the relative fluorescence with respect to growth.

## Supplementary information

Supplementary Information.

## Data Availability

All data analysed in this study are included in this published article and its Supplementary Information files. Raw data are available from the authors on reasonable request.
