# Supplementary material for: Arginine as an environmental and metabolic cue for cyclic diguanylate signalling and biofilm formation in Pseudomonas putida
Source: Sci Rep. 2020 Aug 12;10:13623. doi: 10.1038/s41598-020-70675-x (PMC7423604; doi:10.1038/s41598-020-70675-x)
Supplement: Supplementary file 1 — Supplementary Information. [file 41598_2020_70675_MOESM1_ESM.pdf]

SUPPLEMENTARY INFORMATION

Arginine as an environmental and metabolic cue for cyclic diguanylate signalling and biofilm formation in *Pseudomonas putida*

Laura Barrientos-Moreno[1,2], María Antonia Molina-Henares[1], María Isabel Ramos-González[1], Manuel Espinosa-Urgel[1,*]

[1]*Department of Environmental Protection, Estación Experimental del Zaidín, CSIC, Granada, Spain.*

[2]Present address: *National Biofilms Innovation Centre, Biodiscovery Institute and School of Life Sciences, University of Nottingham, Nottingham NG7 2RD, UK.*

*Address correspondence to Manuel Espinosa-Urgel, manuel.espinosa@eez.csic.es

Table S1. Primers used.

**Primer name**	**Sequence (5´→3´)**[a, b]	**Use**
argT-UpF	ATTGCGGCCGCGGAGCTGAAGAACTGGGTG	Null *argT* mutant construction
argT-UpR	**CATTGCACAGGTGAAACCCGACGTC**CAGGTAACTCCATCGGTACG	
argT-DwF	**TGCCCCGTACCGATGGAGTTACCTG**GACGTCGGGTTTCACCTGT	
argT-DwR	ATTGCGGCCGCACTGCCAGGAAGAAGGTG	
artJ-UpF	ATTGCGGCCGCCGCGACGGTGACGTGATCTC	Null *artJ* mutant construction
artJ-UpR	**GCAAGGGCGCGCCGGTCAGGGCGGA**GTATTGCTCCGTTAGCGGTGG	
artJ-DwF	**CCAGCCACCGCTAACGGAGCAATAC**TCCGCCCTGACCGGCGCG	
artJ-DwR	ATTGCGGCCGCGATGCAGGCCGCGACCAT	
PP_3593-UpF	ATTGCGGCCGCCAAGACCTGCAGAACGATCTG	Null *occT* mutant construction
PP_3593-UpR	**CTGGGCGTTCGCACAACTTCCTGAAGA**CTCGCACCTCATTGCACTCT	
PP_3593-DwF	**TAGCAAGAGTGCAATGAGGTGCGAG**TCTTCAGGAAGTTGTGCGAACG	
PP_3593-DwR	ATTGCGGCCGCGAACATCGGTTCCTTGGTTACG	

[a] Restriction sites inserted in the primer for the cloning strategy are underlined.

[b]Base-complementary between upstream and downstream fragments of the gene to replace are shown in bold.

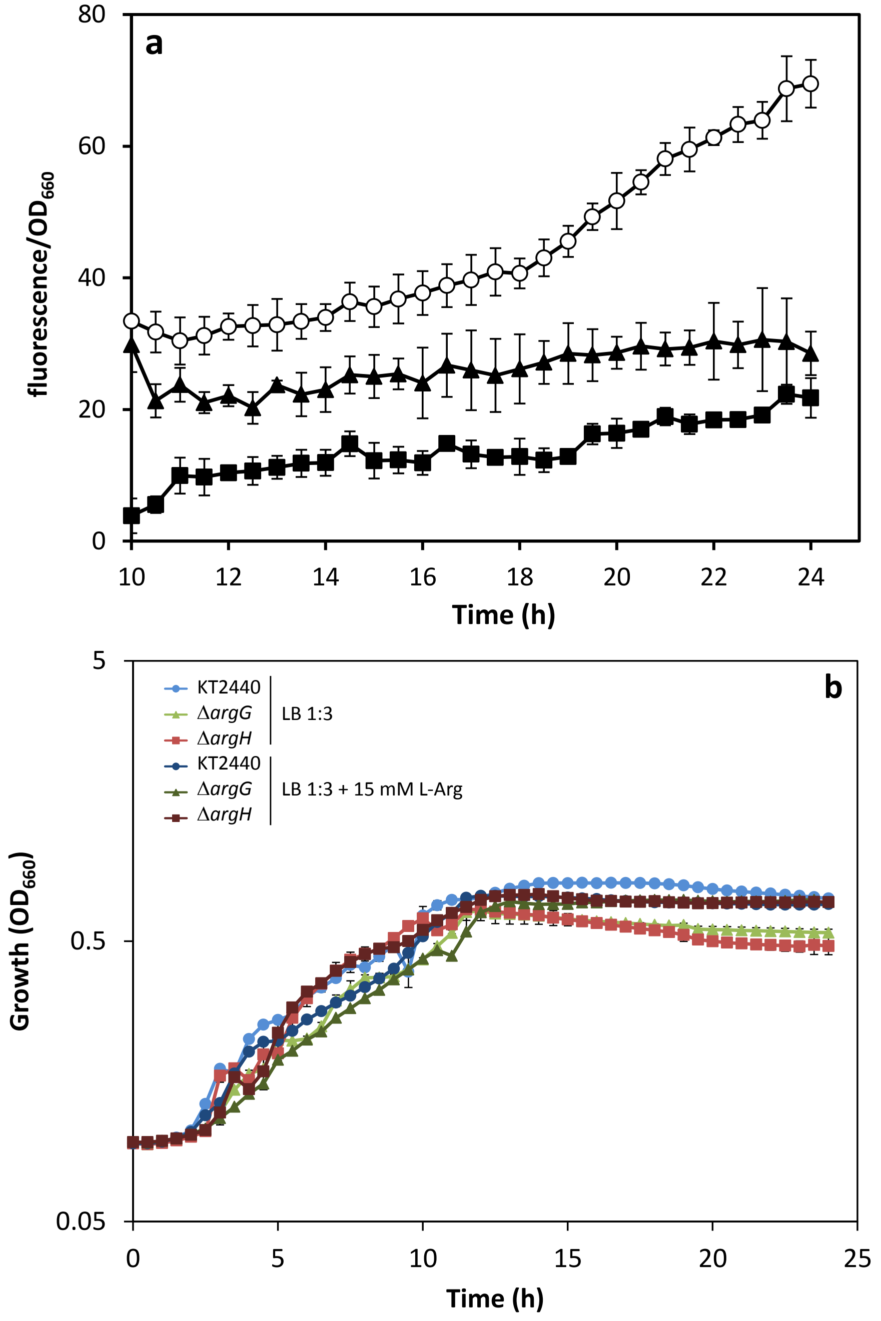

Figure S1. a. Fluorescence-based analysis of c-di-GMP contents in *P. putida* KT2440 (circles), Δ*argG* (triangles) and Δ*argH* (squares) harbouring the biosensor pCdrA::*gfp*^{C} [1], during growth in diluted LB (1:3), using a Synergy Neo2 Biotek fluorimeter. Data are given as fluorescence values corrected by culture growth at each time point. **b.** Growth of the three stains in LB 1:3 and LB 1:3 with 15 mM L-arginine in microtiter plates. Averages and standard deviations of two biological replicates, with three experimental replicates each, are plotted.

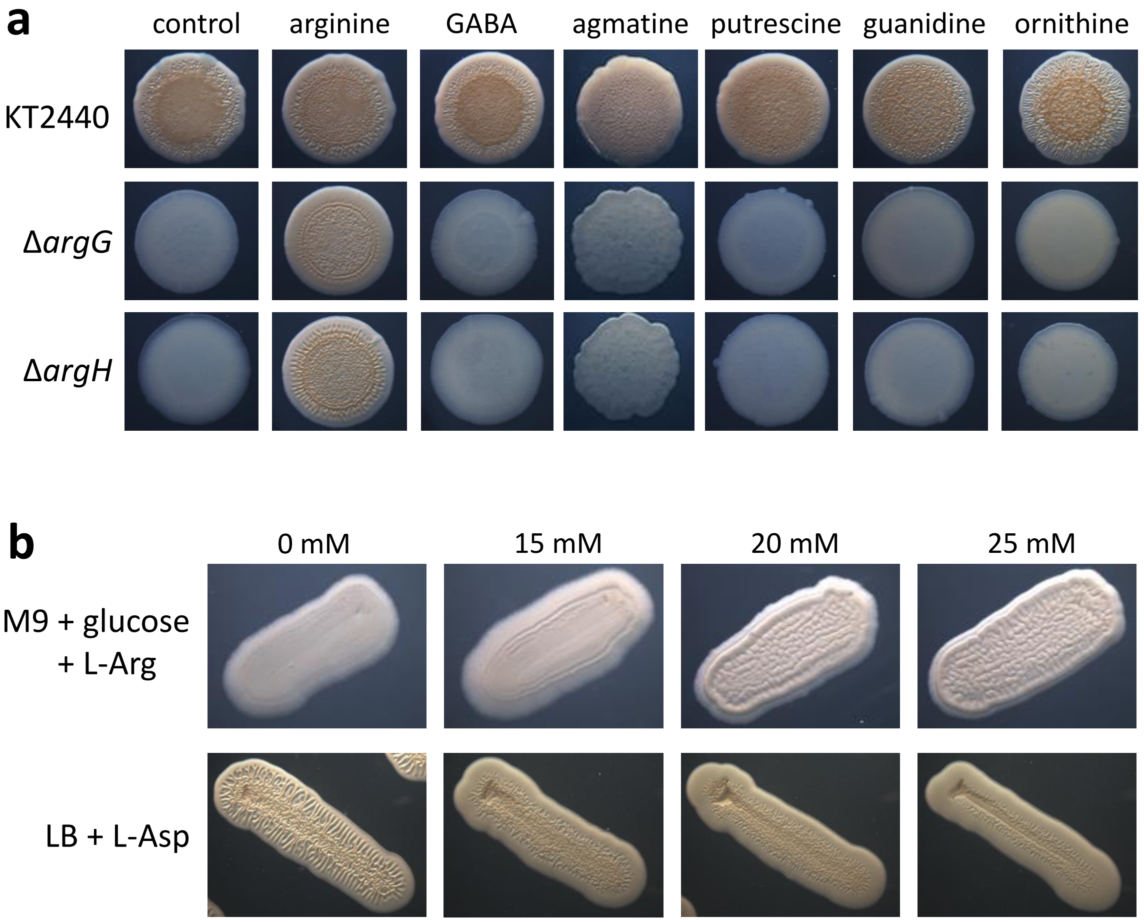

Figure S2. a. Recovery of the crinkly phenotype resulting from overexpression of *cfcR* in Δ*argG* and Δ*argH* mutants harboring pMIR178 (which harbours the gene encoding the response regulator with diguanylate cyclase activity CfcR in multicopy; [2]) by exogenous L-arginine but not by other molecules derived from arginine metabolism. Cultures were spotted on LB plates supplied with the indicated compounds (15 mM), or LB alone as control. Images were taken after 48 h using a Leica M165 FC Stereomicroscope (Leica Microsystems). At that time, the phenotype is fully developed in the wild type. **b.** L-arginine is required for the crinkly colony phenotype of *P. putida* KT2440 carrying pMIR178, whereas L-aspartic acid hampers its development. Images were taken after 48 h of growth in M9 minimal medium with glucose as carbon source or in LB, in the presence of increasing concentrations of the indicated amino acid.

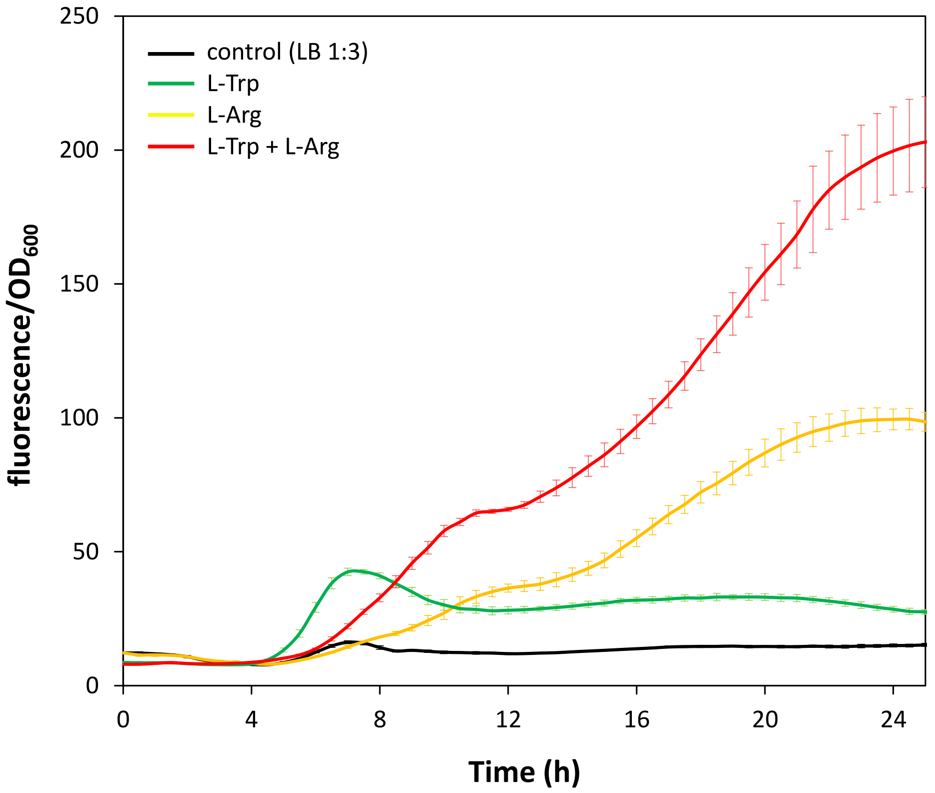

Figure S3. Synergistic effect of L-tryptophan and L-arginine on c-di-GMP levels in *P. putida* KT2440 harbouring pCdrA::*gfp*^{C}. Fluorescence was measured every 30 min. during growth in diluted LB (1:3), without or with amino acid supplementation (15 mM), in a Varioskan Lux fluorimeter. Data are given as fluorescence values corrected by culture growth at each time point and correspond to averages and standard deviations of two biological replicates, with three experimental replicates each.

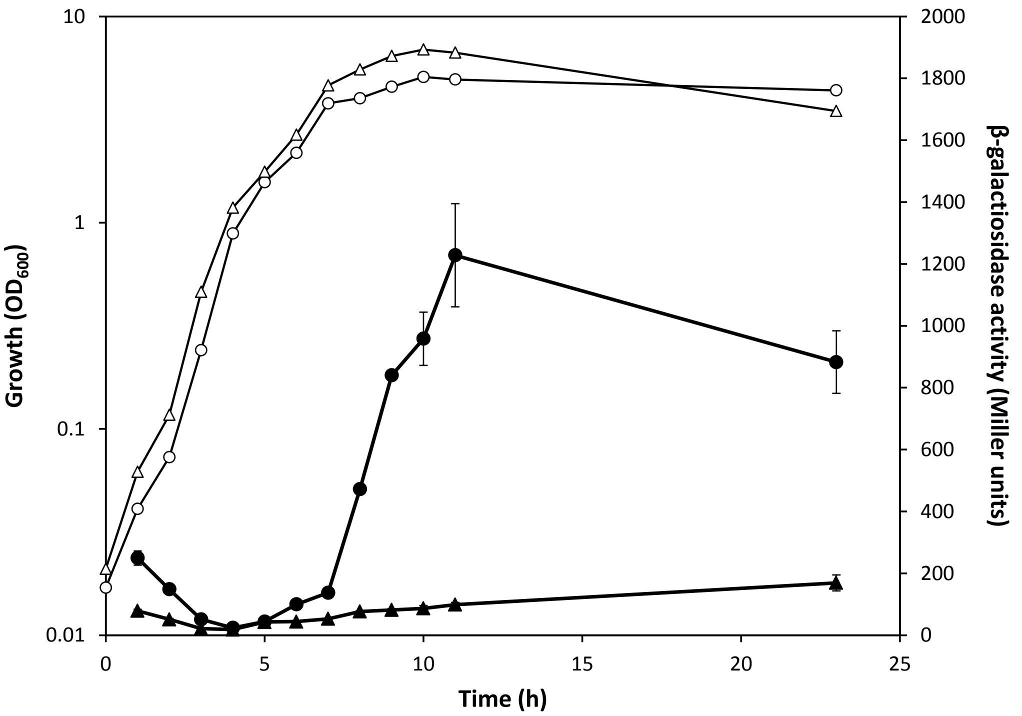

Figure S4. Expression of *pea* is under the control of the stationary phase sigma factor RpoS. Growth (open symbols) and β-galactosidase activity (closed symbols) of KT2440 (circles), and its *rpoS* derivative C1R1 (triangles) [3] harbouring a *pea::lacZ* transcriptional fusion in pMP220 [4] were grown in liquid LB. Data correspond to averages and standard deviations of two experiments with two replicas each. Statistically significant differences between the wild type and C1R1 mutant were observed between 1 and 3 h and from 6 h onward (Student's *t* test: $p \leq 0.05$).

a

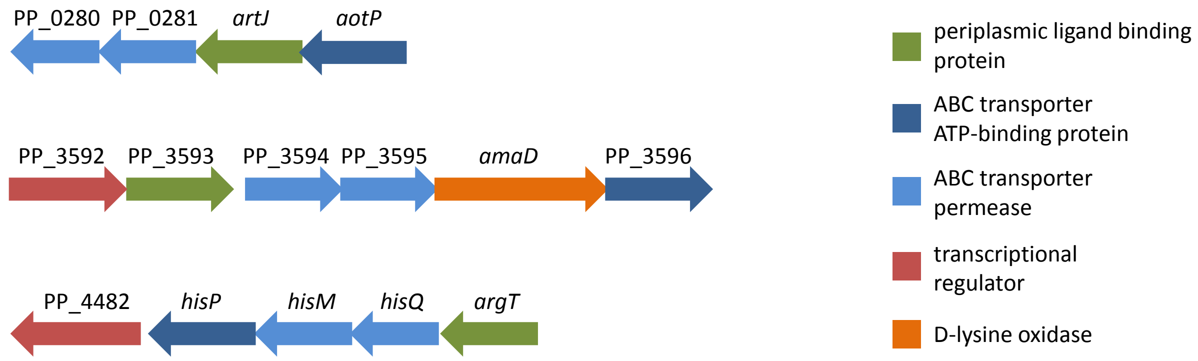

b

```
PP0282        MHTYKKF-LLAAAATLVMSANAMAAEK-LRMGIEAAYPPFNNKDASGNVVGFDKDIGDALCAKMKV
PP3593        MN--KSMALLGACALLL-AG-AASAE-TLRFATEGAYPPFNYVDADNKLHGFDVDITHALCEQMKV
PP4486        MK--K-LALLGALALSVFSLVSQAAEKPLKIGIEAAYPPFAFKQPDGSIAGFDYDIGNALCEEMKT

PP0282        ECSVVTSDWDGIIPALNAKKFDFLVSSLSITDERKQAVDFTNPYYSNKLQFIAPKNVDFKTDKESL
PP3593        ECTLVAQDWEGIIPALMARKYDAIVASMIDTEERRKKIAFTDHYYRTPLTVAVAKDSKIDSAQTDF
PP4486        KCTWVEQEFDGLIPALKVRKIDAILSSMSITDDRKKSVDFTKRYYLTPARLVMKDGTTVSDSLDEL

PP0282        KGKVIGTQRATLAGTYLEDNY--PDVEIKLYDTQENAYLDLVSGRIDGILADKYVQYE-WLKSKDG
PP3593        VGYTVGAQSSSTQAIYAEDVYGKAGADVKLYPTMDEANADLAAGRLDGVIADKFPLHE-WMNKNGG
PP4486        KGKKIGVQRGSIHDRFAKEVLGAKGATVVPYGTQNEIYLDVAAGRLDGTVADATLLEDGFLKTDAG

PP0282        SNFEFKGEPVMDS----DKIGIAVRKGDTKLRDDLNKALAEIKADGTYKKINDKYFPFSIE-----
PP3593        DCCKILG-DVADTK--AD-AAIAVRKDDEALRQRLNTALQQIVANGTYQKIASKYFAFDIYN----
PP4486        KGFAFVGPAFTDAKYFGDGIGIAVRKGDKANVDRINAAIDAIRANGKYKEIESKYFNFDIYGPDSK
```

ArtJ(PP_0282)/OccT(PP_3593):	42% id.; 57% conserv.
ArtJ/ArgT(PP_4486):	42% id.; 58% conserv.
OccT/ArgT:	41% id.; 56% conserv.

Figure S5. a. Genomic context of the loci encoding the three periplasmic binding proteins studied in this work, PP_0282 (*artJ*), PP_3593 (*occT*), and PP_4486 (*argT*). Predicted functions of the adjacent genes are indicated. **b.** Sequence alignment of the three proteins, done with CLUSTALW (https://www.genome.jp/tools-bin/clustalw). Identical residues (id.) in the three proteins are highlighted in yellow, conservative changes (conserv.) in blue, and those identical in two of the proteins in grey. Residues described to interact with L-arginine in related periplasmic binding proteins of *Salmonella* [5] are highlighted in green. Genome and sequence data, and functional annotation of proteins were obtained from the *Pseudomonas* genome database (www.pseudomonas.com; [6]).

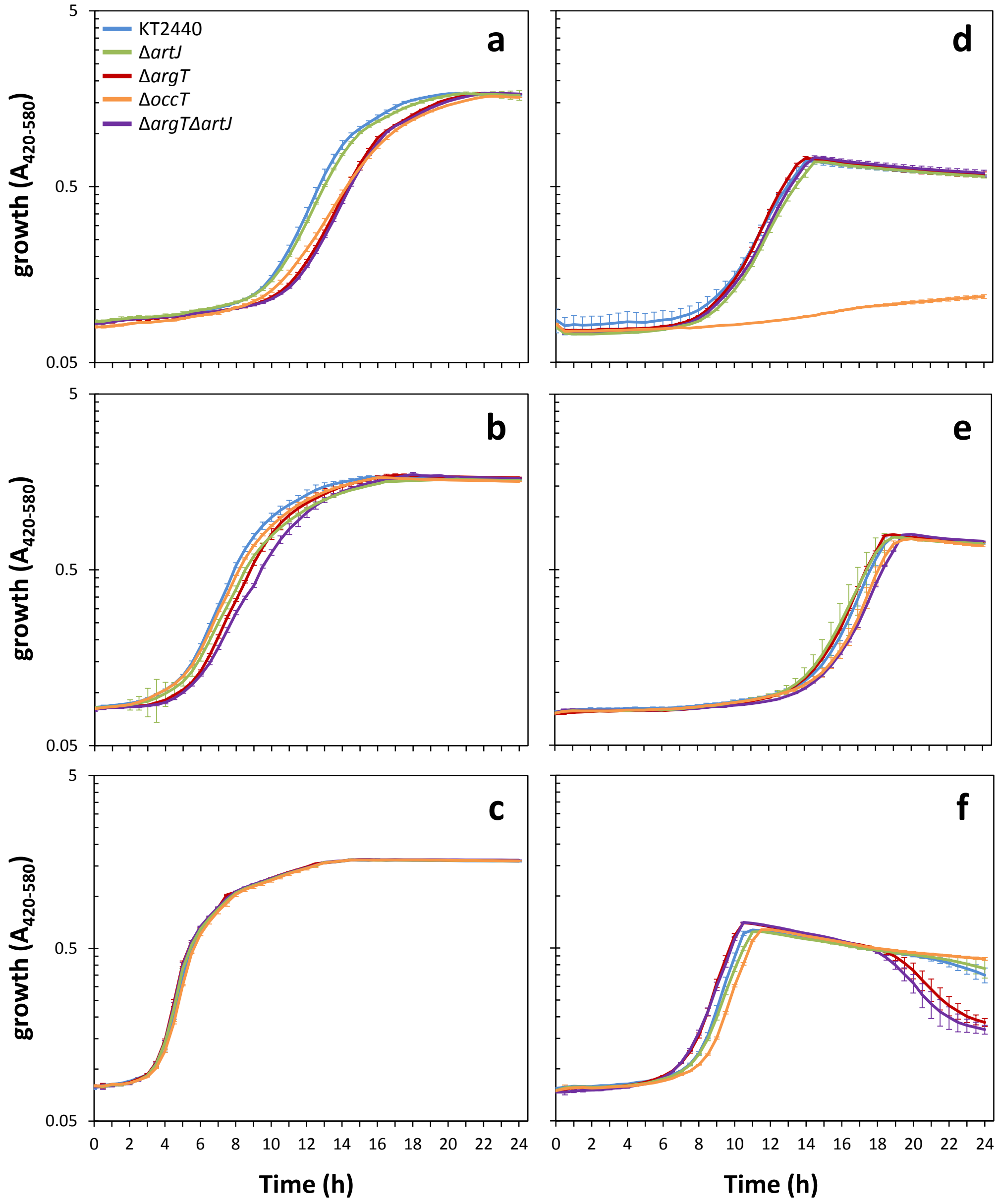

Figure S6. Influence of substrate binding proteins on basic amino acid utilization. Growth of *P. putida* KT2440 (blue lines) and the Δ*argT* (crimson lines), Δ*artJ* (green lines), Δ*occT* (orange lines), and Δ*argT*Δ*artJ* (purple lines) mutants was analyzed in M8 minimal medium with glucose as carbon source and 10 mM L-lysine (**a**), L-ornithine (**b**), or L-histidine (**c**) as nitrogen sources, and in M9 with 10 mM L-lysine (**d**), L-ornithine (**e**), or L-histidine (**f**) as carbon sources. Strains were grown in a Bioscreen C MBR apparatus at 30°C with shaking during 24h in 100-well plates. Absorbance in the 420-580 nm range was measured every 30 min. Averages and standard deviations of one representative experiment per condition, with three technical replicas each, are shown. Color codes are the same as in panel **a** in all cases.

REFERENCES

1. Rybtke, M. T. *et al.* Fluorescence-based reporter for gauging cyclic di-GMP levels in *Pseudomonas aeruginosa*. *Appl. Environ. Microbiol.* **78,** 5060–5069 (2012).
2. Ramos-González, M. I. et al. Genetic dissection of the regulatory network associated with high c-di-GMP levels in *Pseudomonas putida* KT2440. *Front. Microbiol.* **7,** 1093 (2016).
3. Ramos-González, M. I. & Molin, S. Cloning, sequencing, and phenotypic characterization of the *rpoS* gene from *Pseudomonas putida* KT2440. *J. Bacteriol.* **180,** 3421–3431 (1998).
4. Molina-Henares, M. A., Ramos-González, M. I., Daddaoua, A., Fernández-Escamilla, A. M. & Espinosa-Urgel, M. FleQ of *Pseudomonas putida* KT2440 is a multimeric cyclic diguanylate binding protein that differentially regulates expression of biofilm matrix components. *Res. Microbiol.* **168,** 36–45 (2017).
5. Stamp, A.L., *et al.* Crystallographic and microcalorimetric analyses reveal the structural basis for high arginine specificity in the *Salmonella enterica* serovar Typhimurium periplasmic binding protein STM4351. *Proteins* **79,** 2352–2357 (2011).
6. Winsor, G. L. *et al.* Enhanced annotations and features for comparing thousands of *Pseudomonas* genomes in the *Pseudomonas* genome database. *Nucleic Acids Res.* **44,** D646–D653 (2016).
